# Rare Anomaly of Common Bile Duct in Association with Distal Cholangiocarcinoma

**DOI:** 10.1155/2018/8351913

**Published:** 2018-12-17

**Authors:** Arun Pandey, Rajesh Mandal, Paleswan Joshi Lakhey

**Affiliations:** Department of Gastrointestinal and General Surgery-Tribhuvan University Teaching Hospital, Institute of Medicine, Kathmandu, Nepal

## Abstract

Duplication of common bile duct is a rare entity and its association with distal cholangiocarcinoma is extremely rare. It represents failure of regression of the embryological double biliary system. Here, we describe the diagnostic and therapeutic challenges of a type I variant of the extrahepatic bile duct duplication coexistent with distal cholangiocarcinoma that was diagnosed intraoperatively while treating with Whipple procedure for distal cholangiocarcinoma.

## 1. Introduction

Biliary tree anomalies are quite common. Approximately 42% of the general population has some anatomical variation in the biliary tree [1]. Biliary tree anomalies have been reported in association with multiple disorders like recurrent pancreatitis, cholelithiasis, cholangitis, and biliary malignancy [2]. Accurate delineation of biliary tree anatomy is essential for planning a proper surgical treatment and preventing postoperative complications. Duplication of the extrahepatic bile duct is a rare anomaly of the biliary system [2, 3]. Distal cholangiocarcinoma involves malignancy of the distal common bile duct.

## 2. Case Report

A 74-year-old male presented with jaundice for one month associated with generalized pruritus, epigastric pain, anorexia, and weight loss. On clinical examination, the patient was thin built and icteric with palpable gall bladder.

Liver function test was consistent with obstructive jaundice with total bilirubin of 154 *μ*mol/l, direct of 152 *μ*mol/l, SGOT of 147 U/l, SGPT of 67 U/l, and ALP of 731 U/l.

Transabdominal ultrasonography revealed hepatomegaly, moderately dilated IHBD and CBD, and distended gall bladder.

Contrast-enhanced CT of the abdomen with pancreas specific protocol revealed enhancing soft tissue density in the distal common bile duct with upstream dilatation of the CBD, CHD, and IHBD.

**Figure 1:**
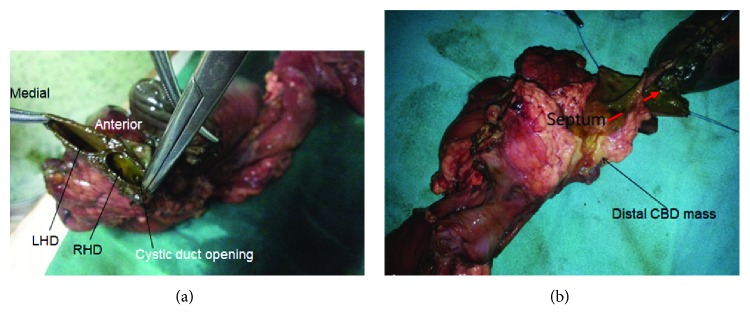
(a) The cystic duct was communicating with the right main extrahepatic duct (findings consistent with type 1 extrahepatic bile duct duplication). (b) Distal CBD mass with septum dividing the CHD and CBD incompletely.

With the diagnosis of distal cholangiocarcinoma, the patient underwent Whipple's pancreaticoduodenectomy. After dividing the common hepatic duct, two openings were appreciated indicating that the division of common hepatic duct occurred at the confluence. However, on further evaluation, it was appreciated that the confluence was intact and it was the septum in the CHD that was giving the false impression. On detailed evaluation of the resected specimen, a septum extending from the hilum with cystic duct opening on the right side (Figure 1(a)) to the distal end of the bile duct was appreciated, suggesting duplication of the common bile duct. However, the septum was not extending up to the papilla (Figure 1(b)).

**Figure 2:**
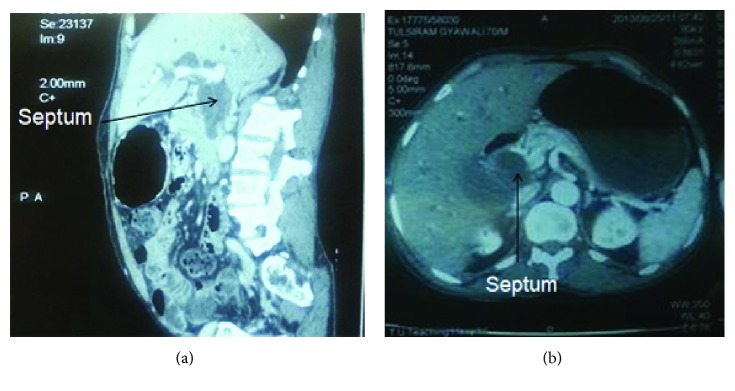
(a, b) CECT of the abdomen: the septum in the CBD extending from the hilum to distal bile duct.

This anatomical variation was not appreciated in the imaging preoperatively. A retrospective evaluation of the cross-sectional imaging revealed an incomplete septum extending from the hilum to the distal end of the common bile duct (Figures 2(a) and 2(b)).

## 3. Discussion

Extrahepatic bile duct duplication is a rare congenital anomaly [4]. There are 5 types of extrahepatic bile duct duplication and type V is the least common [4]. Type 1 represents a septum in the extrahepatic bile duct.

Our case did not resemble type I extrahepatic bile duct duplication exactly, and the septum was extending almost up to the hilum. Thus, it can be considered as a variant of type I.

Extrahepatic bile duct duplication coexistent with pancreaticobiliary malignancy has been reported. Based on a review of the Japanese medical literature, Yamashita et al. reported gastrointestinal cancers in 25% of 47 cases [2]. Cancers of the biliary system were common when the pancreaticobiliary maljunction opened into the second portion of the duodenum or the pancreatic duct [2]. However, pancreaticobiliary maljunction was not identified in our case.

The coexistence of extrahepatic bile duct septum with distal cholangiocarcinoma contributed to a challenging diagnosis and management strategy in our case. CECT scan finding of the septum in the CBD was analysed postoperatively as it was not appreciated preoperatively. Intraoperatively, the septum was seen in the CBD almost extending up to the hilum which was confused as if the CBD was transected high up to the hilum but communicating channel was confirmed by passing a right-angled forceps. Preoperative diagnosis of extrahepatic bile duct duplication is essential for proper surgical planning and can help avoid potential common bile duct injury intraoperatively. In our case, the septum was split and hepaticojejunostomy was done. Since ERCP is an invasive technique and might lead to serious complications, MRCP is preferred over ERCP [4–7]. For type 1 cases, it is harder to be identified. Only 4.2% type 1 cases can be diagnosed preoperatively [5]. Preoperative imaging sometimes may escape diagnosis, mostly diagnosed with intraoperative cholangiogram, ERCP, or MRCP. Obtaining the MDCT images using the MinIP technique plays a decisive role in the preoperative diagnosis and therapeutic planning [8].

**Figure 3:**
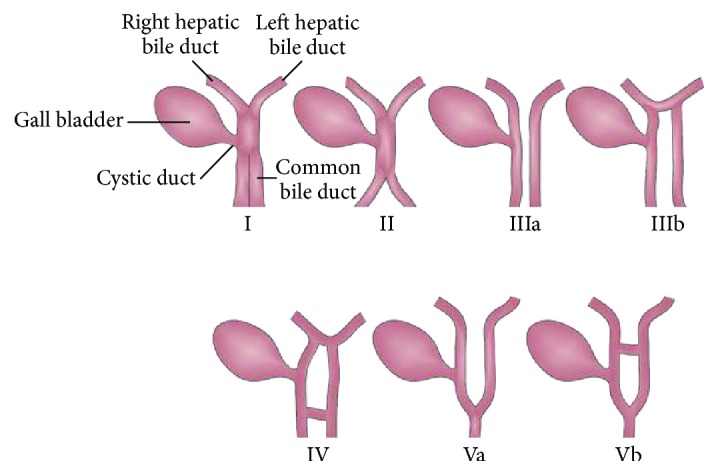
Types of bile duct duplication.

In our case, biliary drainage was achieved by Whipple procedure. This variant of bile duct duplication associated with distal cholangiocarcinoma has not been described in the literature. Our patient had an uneventful postoperative course and followed-up up to 2 years (Figure 3).

## 4. Conclusion

Although biliary tree anomalies are quite common, duplication of common bile duct is a rare entity and its association with distal cholangiocarcinoma is extremely rare. Multiple disorders like recurrent pancreatitis, cholelithiasis, cholangitis, and biliary malignancy are associated with biliary tree anomalies. Preoperative imaging sometimes may escape diagnosis. Intraoperative cholangiogram, ERCP or MRCP, and MDCT images using the MinIP technique can help in the diagnosis. Management of bile duct duplication is according to the associated pathology.
